# Cyclic AMP efflux inhibitors as potential therapeutic agents for leukemia

**DOI:** 10.18632/oncotarget.8986

**Published:** 2016-04-25

**Authors:** Dominique R. Perez, Yelena Smagley, Matthew Garcia, Mark B. Carter, Annette Evangelisti, Ksenia Matlawska-Wasowska, Stuart S. Winter, Larry A. Sklar, Alexandre Chigaev

**Affiliations:** ^1^ University of New Mexico Comprehensive Cancer Center, Albuquerque, NM, USA; ^2^ University of New Mexico Center for Molecular Discovery, Albuquerque, NM, USA; ^3^ Department of Pathology, University of New Mexico Health Sciences Center, Albuquerque, NM, USA; ^4^ Department of Pediatrics, University of New Mexico Health Sciences Center, Albuquerque, NM, USA

**Keywords:** cyclic AMP, drug repurposing, efflux, evasion of apoptosis, leukemia

## Abstract

Apoptotic evasion is a hallmark of cancer. We propose that some cancers may evade cell death by regulating 3′-5′-cyclic adenosine monophosphate (cAMP), which is associated with pro-apoptotic signaling. We hypothesize that leukemic cells possess mechanisms that efflux cAMP from the cytoplasm, thus protecting them from apoptosis. Accordingly, cAMP efflux inhibition should result in: cAMP accumulation, activation of cAMP-dependent downstream signaling, viability loss, and apoptosis. We developed a novel assay to assess cAMP efflux and performed screens to identify inhibitors. In an acute myeloid leukemia (AML) model, several identified compounds reduced cAMP efflux, appropriately modulated pathways that are responsive to cAMP elevation (cAMP-responsive element-binding protein phosphorylation, and deactivation of Very Late Antigen-4 integrin), and induced mitochondrial depolarization and caspase activation. Blocking adenylyl cyclase activity was sufficient to reduce effects of the most potent compounds. These compounds also decreased cAMP efflux and viability of B-lineage acute lymphoblastic leukemia (B-ALL) cell lines and primary patient samples, but not of normal primary peripheral blood mononuclear cells. Our data suggest that cAMP efflux is a functional feature that could be therapeutically targeted in leukemia. Furthermore, because some of the identified drugs are currently used for treating other illnesses, this work creates an opportunity for repurposing.

## INTRODUCTION

Apoptosis serves as a natural barrier to cancer development, and targeting this cancer hallmark represents an indispensable therapeutic strategy [1]. Apoptosis can be induced *via* two major pathways, extrinsic and intrinsic, and in acute myelogenic leukemia (AML) the latter can be directly triggered by elevation of cAMP, which acts synergistically with first-line antileukemic agents [2]. This creates a unique situation, where an additional targetable pathway, previously unexploited by traditional chemotherapeutics, may exist in AML cells [2].

The effect of intracellular cAMP (icAMP) elevation is tissue/cell specific. In certain tumors, including pituitary, adrenocortical and thyroid adenomas and carcinomas, the cAMP/protein kinase A (PKA) pathway provides signals required for tumor development and/or cell survival. In leukemias/lymphomas, cAMP elevation can be pro-apoptotic, whereas in leukocytes/macrophages it is reported to be anti-apoptotic (see Tables 1 and 2 in ref. [3], [4]). Additionally, cAMP can have both pro- and anti-apoptotic activity within the same cell depending upon experimental conditions. icAMP compartmentalization may also contribute to the complexity of signaling [5]. Nonetheless, a significant body of literature suggests that modulating the cAMP pathway provides a number of promising targets for treating leukemia [6].

**Table 1:** Hit compounds identified in the screen for inhibition of cAMP efflux

Hit compound	Structure	Notes
**Artesunate**	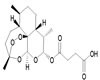	In 2007, FDA approved investigational new drug protocol #76,725 entitled “Intravenous Artesunate for Treatment of Severe Malaria in the United States”
**Dihydroartemisinin**	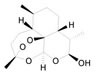	Artesunate is hydrolysed to its active metabolite dihydroartemisinin. Along with artemisinin currently used as antimalarial drugs in Asia.
**Clioquinol**	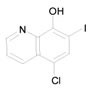	Clioquinol (Iodochlorhydroxyquin) is the FDA approved antifungal and antiprotozoal drug. A phase I trial for clioquinol in patients with hematologic malignancies has been reported [46]
**Cryptotanshinone**	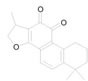	A major tanshinone isolated from Salvia miltiorrhiza. It was shown to inhibit cancer cell proliferation [47]
**Parthenolide**	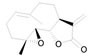	A sesquiterpene lactone from Tanacetum parthenium. An orally bioavailable parthenolide analog selectively eradicates acute myelogenous leukemia stem and progenitor cells [48]
**Patulin**	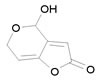	A mycotoxin produced from Penicillium and Aspergillus. Patulin-induced apoptosis in human leukemia cells is mediated through the mitochondrial pathway [49]

**Table 2:** The hematologic cell lines included in the study, their subtype and genetic rearrangements[50]

Cell line	Subtype	Genetic rearrangement	Fusion gene
**U937**	AML	t(10;11)(p12;q14)	PICALM/MLL10(AF10)
**697**	B-ALL	t(1;19)(q23;p13)	TCF3(E2A)/PBX1*
**Nalm-6**	B-ALL	t(5;12)(q33;p13)	ETV6/PDGRFB
**Sup-B15**	B-ALL	t(9;22)(q34;q11)	P190 BCR/ABL1
**Reh**	B-ALL	t(12;21)(q13;q22)	ETV6(TEL)/RUNX1(AML1)
**RS4;11**	B-ALL	t(4;11)(q21;q23)	MLL/MLLT2(AF4)
**MHH-Call 3**	B-ALL	t(1;19)(q23;p13)	TCF3(E2A)/PBX1*

* – Two cell lines were shown to have identical genetic rearrangement and a fusion gene.

AML (IPC-81) and multiple myeloma cells undergo rapid apoptosis after cAMP elevation [7, 8]. In S49 T-cell lymphoma cells, apoptosis can be induced through a cAMP/PKA-dependent pathway [9]. Increasing icAMP by using cAMP analogues [10], *adenylyl cyclase (*AC) stimulators [11], or phosphodiesterase (PDE) inhibitors [12, 13] has been a focus of cancer therapeutics research [3, 14]. This approach has been supported by the fact that the concentration of cyclic nucleotides is elevated in the plasma and urine of individuals with certain leukemias [15, 16], and in some cases these levels correlate with disease progression [16]. In this report, we hypothesize that one mechanism for malignant cell apoptotic evasion could be active efflux of cAMP [14]. Rather than relying on PDEs to degrade icAMP, active cAMP removal from the cytoplasm can provide a survival advantage. We envision that cAMP efflux prevents an elevation of icAMP that could trigger up-regulation of the Bcl-2 interacting mediator of cell death (Bim/BCL2L11) protein [2, 17], or down-regulation of the myeloid cell leukemia 1 (Mcl-1) protein [8, 18]. Inhibition of cAMP efflux alone should be sufficient to selectively trigger death in cells that rely on this anti-apoptotic mechanism for survival. To test this idea, we decided to identify drug-like compounds that are capable of blocking cAMP efflux.

To identify “inhibitors of cAMP efflux” (ICE), we developed and validated a novel assay for the detection of cAMP efflux (in press), using a well characterized model for AML known to efflux cAMP through ABCC4, the multidrug resistance-associated protein-4 (MRP4) transporter [19]. Next, we screened libraries composed of biologically active substances and off-patent drugs. We validated the “hits” in secondary assays that assessed the compound effects on cell signaling, viability and apoptosis. The ICE were also tested for their effects on cAMP efflux inhibition and viability in B-lineage acute lymphoblastic leukemia (B-ALL) cells, primary B-ALL bone marrow patient samples, and healthy human primary blood mononuclear cells (PBMCs). The most promising compounds showed dose-dependent, selective inhibition of leukemic cells *vs*. PBMCs. Our hypothesis was further supported by measurements of cAMP-dependent activation of downstream effectors after exposure to ICE compounds. Because several identified ICE are FDA-approved drugs, our studies provide a potential path for drug repurposing against leukemias.

## RESULTS

### A screen for ICE identifies six potentially active compounds

To identify ICE, we took advantage of a model system where cAMP efflux is well studied: U937 cells, which can actively extrude cAMP into extracellular media [19]. In these cells, a rapid increase of cAMP efflux can be triggered through the elevation of the icAMP concentration using G-alphaS GPCR-specific ligands, blocking PDE-dependent cAMP hydrolysis, and by other pharmacological manipulations. The cAMP efflux is ATP- and MRP inhibitor-dependent, and shRNA knockdown has shown that the cAMP efflux is mediated by MRP4/ABCC4. Moreover, an increase of icAMP was sufficient to induce differentiation of U937 and other AML cell lines [19].

**Figure 1:**
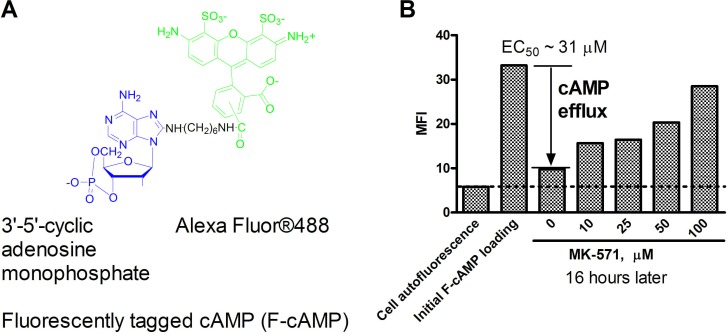
Effect of MK-571 (MRPs selective inhibitor) on efflux of a fluorescently tagged cAMP (F-cAMP) **A.** Structure of the fluorescent cAMP analog. **B.** U937 cells loaded with F-cAMP were incubated overnight in the presence of increasing concentrations of MK-571 at 37°C. The EC_50_ for cAMP efflux blocking was determined using a variable slope sigmoidal dose response equation with “bottom” constraint equal to cell autofluorescence and “top” constraint equal to the specific fluorescence. A representative experiment of three experiments is shown.

To study cAMP efflux, we loaded U937 cells with fluorescently tagged cAMP (F-cAMP). MK-571, (MRPs selective inhibitor) [19], was used as a positive control. Figure 1 shows that MK-571 down-modulated cAMP efflux in a dose-dependent manner, with EC_50_ ~30 μM. This value was very close to the IC_50_ previously reported for MRP4/ABCC4 [20]. Thus, the fluorescent tag (Alexa Fluor^®^488) did not significantly affect the ability of cells to efflux cAMP nor of MK-571 to block this process.

These experiments served as the basis for a screen to identify compounds that could block cAMP efflux. F-cAMP-loaded U937 cells were screened against compounds from the Prestwick Chemical Library (~1200 previously FDA-approved drugs) and the SPECTRUM Collection (2320 compounds - 60% drugs, 25% natural products, 15% bioactive components). The screen identified 51 hits, which were tested in dose-response to validate their activities (data not shown), yielding 7 potentially active ICE compounds: artemisinin, clioquinol, quinalizarin, harmalol, cryptotanshinone, parthenolide, and patulin. Three additional structurally-related compounds (dihydroartemisinin, artemether, artesunate) were also included for evaluation in secondary assays. Based on further validation, only artesunate, clioquinol, cryptotanshinone, dihydroartemisinin, patulin, and parthenolide were chosen for extended studies (Table 1). The detailed screening data will be published elsewhere (in press).

### Correlation between ICE ability to block F-cAMP efflux and viability loss

**Figure 2:**
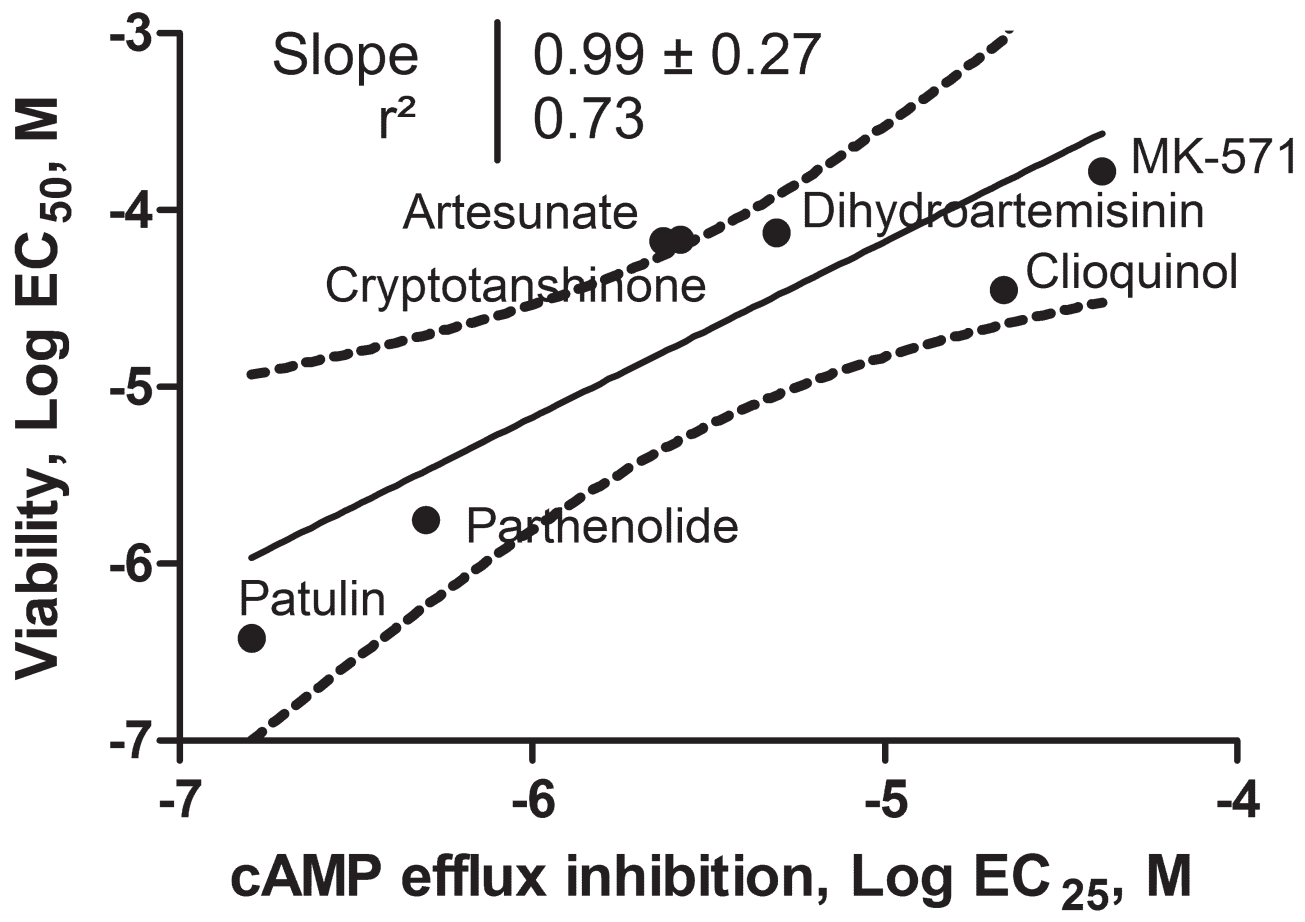
Relationship between ICE ability to block F-cAMP efflux and loss of cell viability U937 cell were incubated overnight in the presence of increasing concentrations of ICE and MK-571 and cell viability was determined as described in **Materials and Methods**. Next, the data were fitted to a sigmoidal dose response equation and the EC_50_ values for cell viability loss were plotted *vs.* EC_25_ determined for F-AMP efflux inhibition. The EC_25_ was equivalent to a two standard deviation cut-off that was used for a primary compound screening hit determination criteria. The data were fitted to a linear regression equation. The 95% confidence interval, a square of Pearson's correlation coefficient and a slope of the line are shown.

Next, we compared ICE efficacy for blocking F-cAMP efflux with their ability to trigger loss of cell viability (Figure 2). A strong positive correlation (r^2^ = 0.73) has been observed. The values for all six ICE compounds and MK-571 control ranked in the same order and were located within 95% confidence interval. Moreover, the slope of the regression line was equal to 1. This suggests that the relative potency of ICE in affecting cell viability varies in parallel with the ability to block F-cAMP efflux.

### CREB/AFT-1 phosphorylation in response to ICE

**Figure 3:**
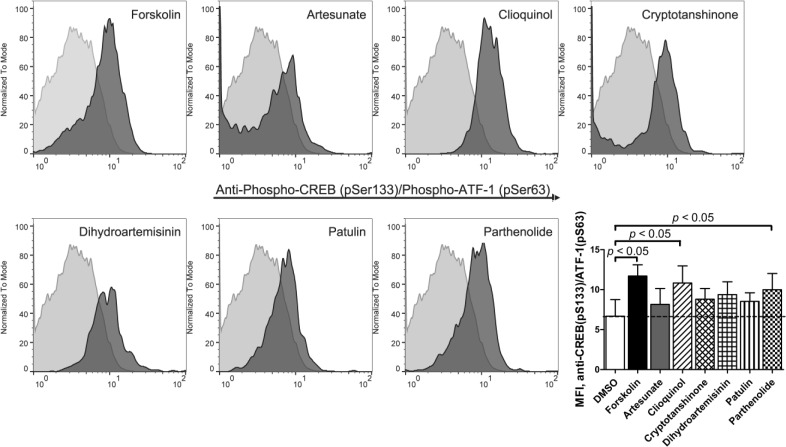
Binding of anti-phospho-CREB/AFT-1-specific antibody in response to ICE U937 cells were treated for 1 hour with 20 μM ICE compounds or forskolin (positive control), or DMSO (vehicle, negative control). Next, cells were fixed, permeabilized and stained with primary labelled anti-CREB (pSer133) / ATF-1 (pSer63) monoclonal antibody. Histogram overlays from one representative experiment show negative control events (light grey) and compound-treated events (dark grey). Bar graph shows MFI ± SEM (standard error of the mean) for four independent experiments. Statistical significance was determined by one-way ANOVA with repeated measures using a Dunnett post-test to compare treated samples to DMSO control values (*p* < 0.05).

Next, to evaluate whether reducing cAMP efflux would result in an elevation of cytoplasmic cAMP-dependent cell signaling, we studied the effects of ICE on phosphorylation of cAMP-responsive element-binding protein (CREB; Ser133) and activating transcription factor-1 (ATF-1; Ser63), classical cAMP effectors that activate target genes through cAMP response elements (CRE). This pathway is also directly implicated in cAMP-induced apoptosis in leukemia [2]. All studied compounds showed increased binding of anti-CREB (pS133) / ATF-1 (pS63) specific antibodies as compared to vehicle control (Figure 3). For two compounds (clioquinol and parthenolide), the binding of antibodies was comparable to the adenylate cyclase stimulator forskolin positive control. Thus, ICE compounds can stimulate CREB/AFT-1 phosphorylation.

### VLA-4 deactivation in response to ICE

**Figure 4:**
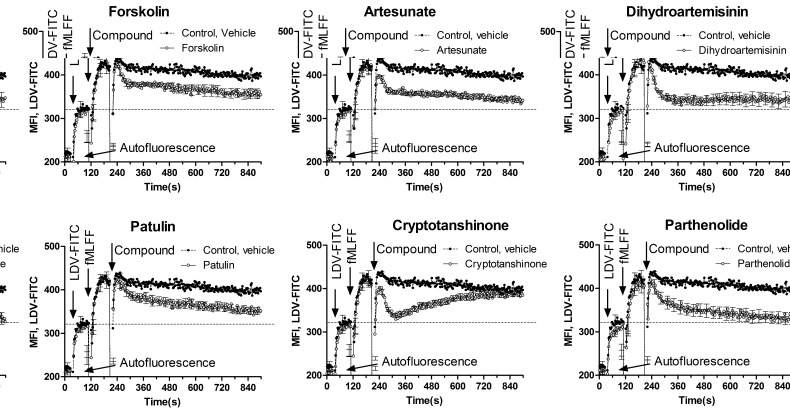
Dissociation of the VLA-4-specific fluorescent ligand in response to ICE U937 cells transfected with a non-desensitizing mutant of FPR1 were maintained in cRPMI at 37°C while the samples were stirred continuously during data acquisition. Cell suspension was sequentially treated with LDV-FITC (first arrow), FPR1-specific ligand for cell activation (fMLFF, second arrow), and ICE compounds or forskolin positive control (40 μM) or DMSO vehicle (third arrow). The use of the non-desensitizing mutant of FPR1 allows for the maintenance of the high affinity VLA-4 state during the course of the experiment (control, vehicle). This allows for monitoring the real-time deactivation kinetics after compound addition. Each line represents MFI ± SEM of three independent experiments. In each experiment, every experimental point represents MFI of several hundred events acquired every second. Bars representing SEM are shown for every 10 s time point. Dashed line represents binding of the LDV-FITC prior to cell activation.

Another signaling pathway that in leukocytes can be triggered by the elevation of cytoplasmic cyclic nucleotides is the conformational deactivation of the Very Late Antigen-4 (VLA-4, alpha4 beta1 integrin), an adhesion molecule implicated in homing and retention of early hematopoietic progenitors in the bone marrow. The elevation of icAMP using G-alphaS GPCR-specific ligands, forskolin and by other pharmacological manipulations results in rapid dissociation of the VLA-4-specific ligand-mimicking probe, LDV-FITC [21]. We studied the effect of ICE on VLA-4 deactivation using the same previously characterized model system (Figure 4). Studied compounds triggered rapid dissociation of LDV-FITC in U937 cells pre-activated through a non-desensitizing mutant of the FPR1. In several experiments, the effects of parthenolide and patulin exceeded the effects of the positive control, forskolin (Figure 4). Cryptotanshinone induced rapid and reversible VLA-4 deactivation that looked similar to the effect of a cell-permeable cyclic nucleotide analog [22]. Thus, ICE compounds were also capable of triggering an integrin de-activation pathway, where the role of elevated cyclic nucleotide concentration is critical [21, 22].

### Adenylyl cyclase inhibition reduces ICE-induced cell viability loss

**Figure 5:**
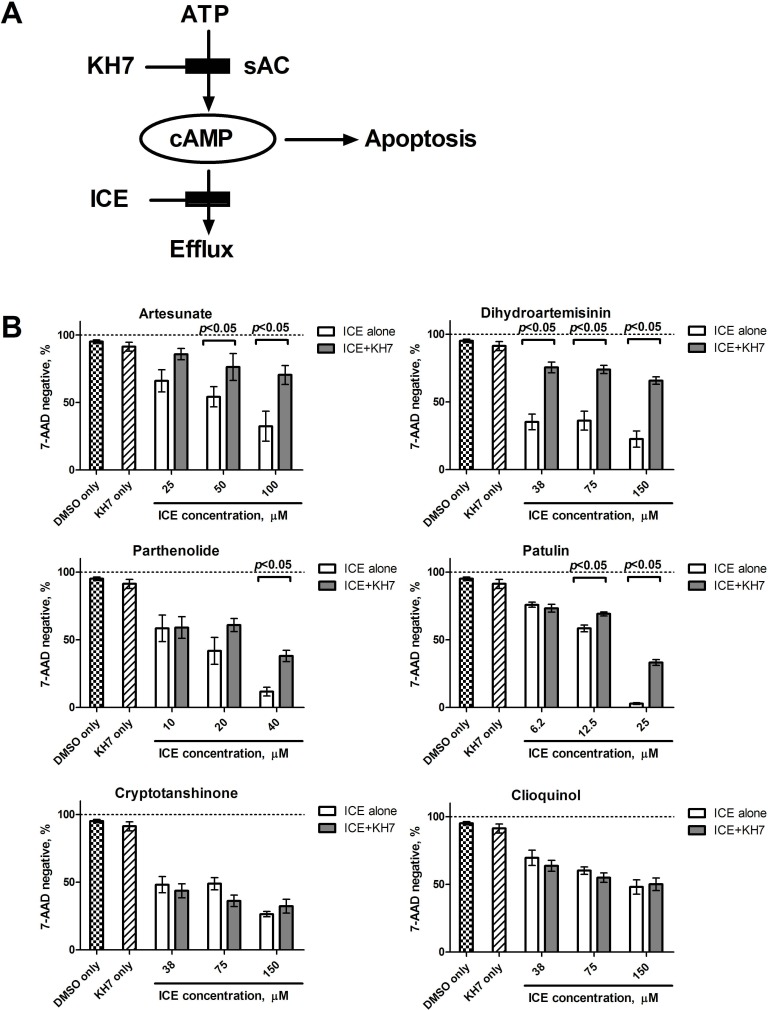
Effect of the selective inhibitor of soluble adenylyl cyclase (KH7) on cell viability loss induced by ICE **A.** Schematics of KH7 action. By blocking synthesis of cAMP, KH7 is expected to decrease overall icAMP accumulation and thus, to protect cells from ICE-induced apoptosis. **B.** U937 cells were treated overnight with increasing concentrations of ICE in the presence or absence of 6 μΜ ΚΗ7. ICE concentrations were chosen based on relative ICE potency. Cell viability was assessed using 7-AAD staining as described in **Materials and Methods**. Control samples were treated with 6 μΜ ΚΗ7 or equal volume of vehicle (DMSO). Graph shows percentage of 7-AAD negative events ± SEM of two independent experiments. Each bar represents mean of five values (*n* = 5). Statistical significance was determined using unpaired *t*-test and significant difference is indicated as (*p* < 0.05) for each pair of KH7 treated and untreated samples.

Because synthesis is a source for icAMP accumulation, and soluble adenylyl cyclase (sAC) is directly implicated in the mitochondrial pathway of apoptosis, we studied how blocking the AC by the selective sAC inhibitor, KH7, influences ICE effects. Based on the data published by Kumar *et al.*, 2009 [23], we expected that blocking cAMP production would reduce ICE potency (Figure 5A). In fact, we detected a significant protective effect of KH7 on the cell viability loss induced by the four most potent ICE compounds (Figure 5B). The lack of effect for less potent ICE could be due to a lesser effect on viability: KH7 displayed the most prominent effect in samples where control viability was far below 50%. Thus, inhibition of sAC activity was sufficient to reduce the effects of the most potent ICE.

Hence, compounds identified in an assay based on blocking cAMP efflux can reduce cell viability, and stimulate two signaling pathways that are each modulated by elevation of icAMP, CREB phosphorylation and VLA-4 deactivation. Moreover, blocking of sAC activity prevented ICE-induced cell viability loss. These data are consistent with an ICE molecular mechanism involving elevation of icAMP. The accumulation of icAMP as the result of decreased cAMP efflux in U937 cells was first demonstrated by Copsel, *et al*. [19].

### Normal primary peripheral blood mononuclear cells (PBMCs) did not significantly efflux cAMP

**Figure 6:**
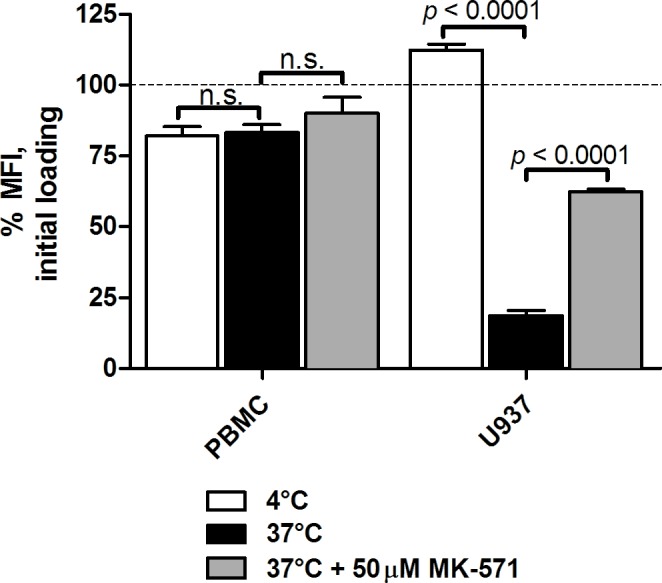
cAMP efflux activity of U937 cells and human PBMCs Cells were loaded with F-cAMP as described in **Materials and Methods**. F-cAMP retention after overnight incubation with ICE in samples incubated at 4°C (passive leakage) or 37°C (active removal), and in the presence of cAMP efflux inhibitor control MK-571, was measured by flow cytometry. Cell autofluorescence was subtracted, and the data were normalized to the fluorescence value at initial staining indicated by dashed line. Data shown is MFI ± SEM from one representative independent experiment conducted in triplicate. Statistical significance was determined by a two-tailed Student's *t*-test, n.s., non-significant.

According to our hypothesis, active cAMP efflux represents a novel apoptosis evasion mechanism that is activated in certain malignant cells, and normal cells should lack this cAMP efflux ability. To test this proposition, we compared F-cAMP efflux in U937 cells and normal PBMCs (Figure 6). After 24 h incubation, PBMCs loaded with F-cAMP retained ~80-90 % of the probe fluorescence when incubated at 37°C or at 4°C, whereas U937 cells lost ~80 % of the probe fluorescence only at 37°C. A significant part of this loss was blocked by incubation with efflux inhibitor MK-571, indicating active participation of ABCC family transporters. No decrease in the probe fluorescence was detected at 4°C, suggesting that cell membrane integrity was well preserved and passive probe leak had not occurred. This result suggests that active cAMP removal cannot be detected in normal PBMCs in the same time frame as in the AML model.

### ICE induce apoptosis of U937 cells

**Figure 7:**
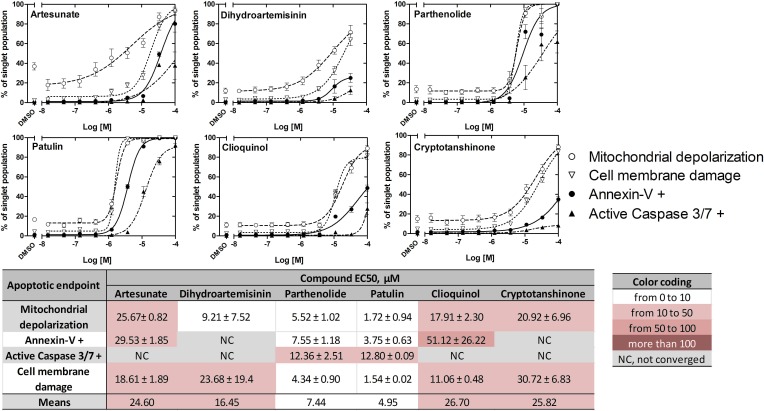
Effects of ICE on apoptosis of U937 cells Cell mitochondrial depolarization, annexin-V binding, caspase 3/7 activation and cell membrane damage are shown as dose response curves and a heat map. Percentage of U937 cells positive for four different apoptosis endpoints was determined as described in **Materials and Methods**. Dose response curves for each endpoint (*n* = 4) after 24 h incubation with ICE display cell viability (%) ± SEM. Data were fit using variable slope sigmoidal dose response equation with no constraints. Heat map shows EC_50_s ± SEM. NC, not converged.

The effect of ICE on U937 cell apoptosis after overnight incubation has been studied using the MultiCyt 4-Plex Apoptosis kit that reports four different apoptosis endpoints: effector caspases 3 and 7 activation, phosphatidylserine surface expression, mitochondrial membrane depolarization and cell membrane integrity. The ICE induced apoptosis in a dose-dependent manner, and the EC_50_ values for all four apoptosis endpoints were determined to range from 2-3 μM to several hundred μM (Figure 7). The relative sensitivity of different apoptosis endpoints reflected different consecutive steps of the intrinsic pathway. Mitochondrial depolarization and membrane damage were the most sensitive while the effector caspase activation was the least. Cryptotanshinone showed a very small effect on annexin-V binding and caspase activation, even at the highest ICE concentrations. These EC_50_s were excluded from further analyses (as indicated by NC).

To simplify analysis, the data were color-coded (heat map) according to the determined EC_50_ values (Figure 7). The most potent compounds (EC_50_ values 1-10 μM) in the apoptosis assay were: patulin, parthenolide, and dihydroartemisinin (white color). In addition, the relative ranking was largely independent on the particular endpoint. Mean EC_50_s calculated for each compound are shown at the bottom of a heat map and indicate relative potency of studied ICE. Thus, U937 cells treated with ICE exhibited a dose-dependent increase in apoptosis.

### ICE decrease viability and show cancer cell specificity

**Figure 8:**
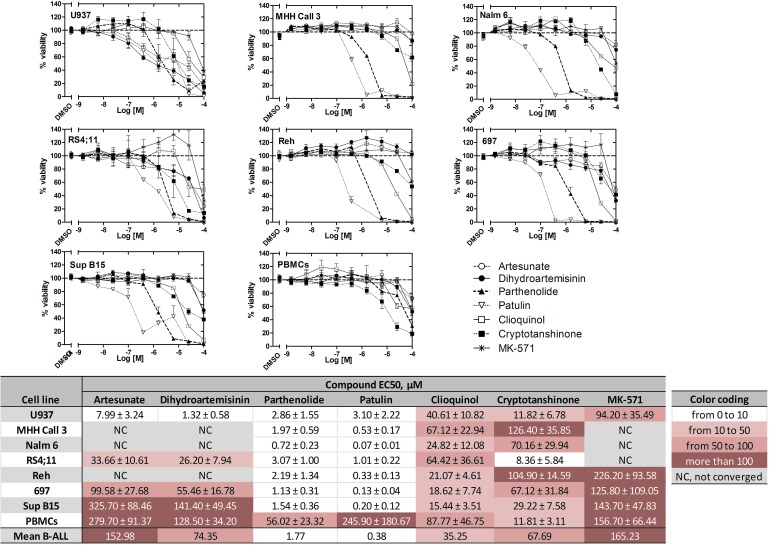
Effects of ICE on cell viability of U937 cells, the B-ALL cell lines 697, Reh, MHH Call 3, RS4;11, Sup B15, and Nalm 6, and normal human PBMCs Dose response curves for each cell type after overnight incubation with ICE display cell viability (%) ± SEM. For U937, *n* = 8; for PBMCs and B-ALL cells, *n* = 6. Data were normalized assuming that negative control (DMSO) was equal to 100% viability. Data were fit using the sigmoidal dose response equation with constraints: top = 100, bottom = 0, hill slope = −1. Table shows EC_50_s ± SEM. NC, not converged.

Next, we tested the effect of ICE on the viability of U937 cells and B-ALL cell lines (Table 2). On U937 cells, several compounds were more potent than the positive control MK-571 (Figure 8). In general, B-ALL cell lines showed more sensitivity than the U937 cells to several of the selected compounds. However, B-ALL lines showed a decreased sensitivity to the two structurally related drugs: artesunate and dihydroartemisinin. The EC_50_ values for the ICE with each B-ALL line ranged from low nanomolar to ~300 μM (Figure 8). It can be noted, however, that the compounds, which consistently decreased viability ranked in the same order across all six B-ALL lines: patulin, parthenolide, clioquinol, cryptotanshinone.

To investigate the potential selectivity of the compounds, we compared ICE effects on cell viability in leukemic and non-malignant cells (PBMCs from healthy volunteers). The EC_50_ values indicated that for the most potent of the tested compounds to be efficacious, PBMCs required a concentration of at least an order of magnitude higher than was necessary for the leukemic cell lines (Figure 8, notice darker color-coding for PBMCs). This difference was expected, since normal PBMCs do not have an established system for cAMP efflux (Figure 6), and therefore should be less sensitive to ICE.

### Leukemic cells have different abilities to efflux cAMP

**Figure 9:**
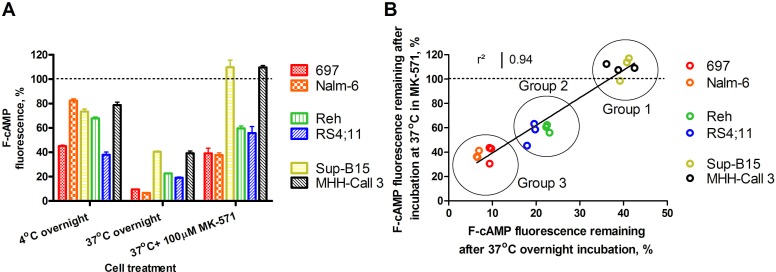
Retention of F-cAMP in hematopoietic cell lines **A.** F-cAMP leakage from B-lineage ALL cell lines after overnight incubation at 4°C, or 37°C, and in the presence of the positive control MK-571. Lower values correspond to higher efflux. Data shown are the mean ± SEM from three independent experiments. Initial F-cAMP loading is indicated by a dashed line. **B.** The correlation between F-cAMP fluorescence remaining in cells incubated at 37°C alone and in the presence of MK-571. See text for details.

To further study cAMP efflux in leukemic cells, the B-ALL cell lines were loaded with F-cAMP, and its efflux from the cells was evaluated. After incubation at 4°C (passive leakage), the cells lost 20-60% of their fluorescence from the initial F-cAMP loading. Incubation at 37°C (active efflux) resulted in F-cAMP removal that ranged from ~60% to ~90%, with no apparent relationship between 4°C and 37°C samples (Figure 9A). For example, Nalm-6 and 697 cells showed dissimilar passive leakage, but both cell lines exhibited similar active F-cAMP removal abilities.

The cell lines also exhibited varied sensitivity to MK-571, a control for transporter-dependent efflux. We found a strong relationship between levels of F-cAMP remaining in cells at 37°C, both alone and in the presence of MK-571. Based on this parameter, cell lines could be stratified into three groups, which indicated correlations between ability to actively efflux cAMP and MK-571 inhibition of cAMP removal. *Group 1:* Sup-B15 and MHH-Call3 removed ~60% of the F-cAMP by active efflux, which was blocked completely by MK-571. *Group 2:* Reh and RS4;11 cells removed 75-80% of the F-cAMP by active efflux, and ~30-40% with MK-571. *Group 3:* 697 and Nalm-6 cells removed > 90% of the F-cAMP by active efflux, and 60-70% with MK-571 (Figure 9B). Thus, the cell lines that effluxed F-cAMP poorly were better inhibited by MK-571, and those that were efficient in the removal of F-cAMP were less inhibited by MK-571. This suggests that for cell lines in Groups 2 and 3, an additional MK-571-insensitive mechanism participated in the removal of F-cAMP.

### The primary cAMP transporter, MRP4/ABCC4, is differentially expressed by leukemic cell lines

**Figure 10:**
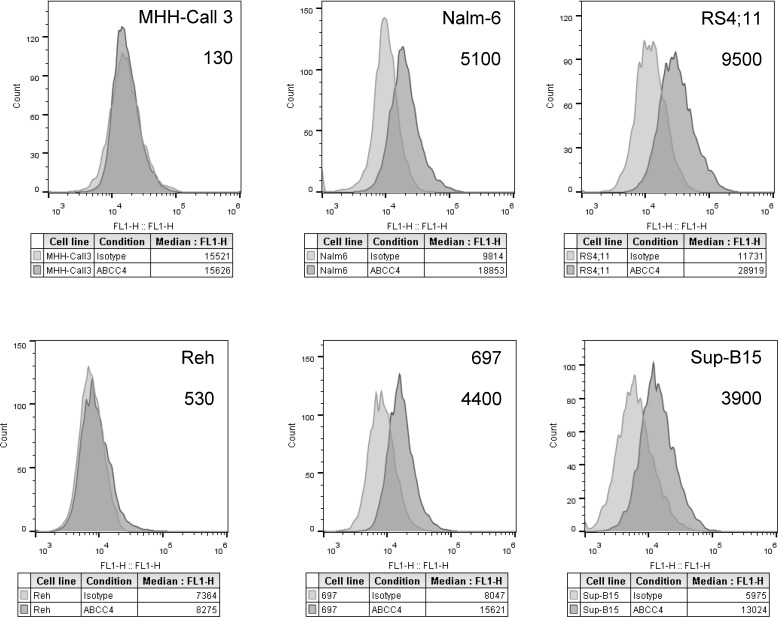
Determination of ATP-binding cassette transporter ABCC4-specific binding sites on B-ALL cell lines Raw histograms of IgG isotype controls (grey) and ABCC4-specific antibody (dark grey) binding to each cell line. Specific binding sites calculated using Quantum™ Simply Cellular^®^ anti-human IgG calibration beads stained with primary anti-ABCC4-specific and secondary fluorescent antibody. Calibration beads allowed mean fluorescence intensity (MFI) values to be converted into non-specific binding sites. Binding site values for IgG primary antibody-bound isotype control cells were subtracted from the ABCC4 non-specific binding site data to calculate the number of ABCC4-specific binding sites per cell. Estimated site numbers are indicated on each panel. A representative experiment of two experiments is shown.

cAMP is primarily released by cells *via* the ABCC4 (MRP4) and ABCC5 (MRP5) transporters [24]. The affinity of ABCC4 for cAMP is ~10-fold greater than that of ABCC5 (k_m_ values of 44.5 μM and 379 μM, respectively) [24, 25]. Therefore, we hypothesized that the presence of ABCC4 on leukemic cells would correlate with their ability to efflux F-cAMP. ABCC4 phenotypes were determined for the B-ALL cell lines, and the number of ABCC4-specific binding sites ranged from ~100 on MHH-Call3 to ~10^4^ on RS4;11 cells (Figure 10). However, no apparent correlation between ABCC4 expression and F-cAMP efflux were detected (compare Figure 9 and Figure 10). This suggests that other transporters and/or mechanisms may play additional roles in leukemic cell removal of cAMP.

### ICE decrease primary ALL patient sample viability

**Figure 11:**
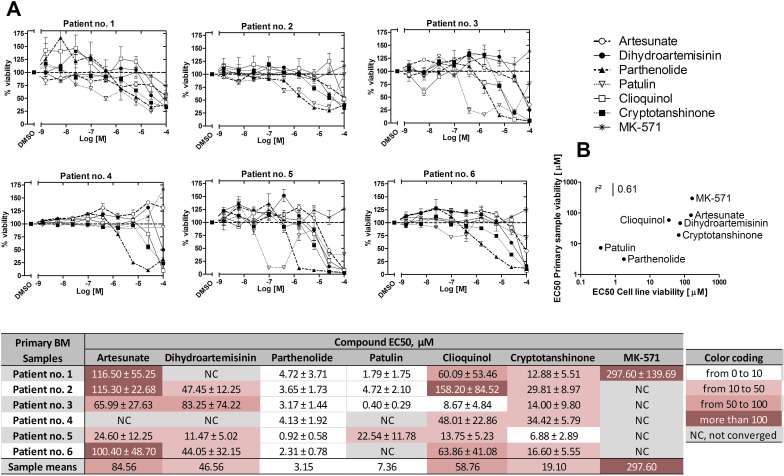
Effects of ICE on cell viability of primary ALL patient samples **A.** Compound dose response curves for each patient sample tested in duplicate. Data were normalized assuming that negative control (DMSO) was equal to 100% viability. Data were fit using the sigmoidal dose response equation with constraints: top = 100, bottom = 0, hill slope = −1. Table shows EC_50_s ± SEM. NC, not converged. **B.** Correlation between mean compound EC_50_ values obtained from B-ALL cell line data (Figure 8) and mean EC_50_ values from primary B-ALL patient samples. The coefficient of determination (r^2^) was calculated from the Pearson correlation coefficient.

To further establish the effectiveness of ICE in reducing cancer cell viability, primary cells from six B-ALL patients (Table 3) were tested *ex vivo*. The results showed dose-dependent decreases in patient sample viability after exposure to the compounds (Figure 11). To verify the relative ranking of ICE, the mean EC_50_ values for each compound were plotted against the mean EC_50_ values determined for all B-ALL cell lines. The result indicated a strong correlation for most patient samples (Figure 11B). This result suggests that the data obtained using cell lines adequately reflect the relative compound potency detected in patient samples, and therefore, cell lines can be used to test ICE derivatives in future medicinal chemistry applications.

**Table 3:** Genotypic and phenotypic profile of B-ALL patient samples

Patient	Age	Gender	WBC	Ploidy	Phenotype	Risk	Cytogenetics/FISH
**124-12**	4	F	2.9			STD	46, XX,del(9)(p13),der(19)t(1;19)(q23;p13.3)[10]/46,idem,add(5)(p13),add(9)(p21),del(10)(q23)[2]/46,idem, t(1;13)(p13;q14)[3]/46, XX[5]
238-13*	18	M	629.2	diploid	CD34+ CD19+ CD22dim	VHR	46, XY,t(4;11)(q21;q23)[cp6]/49, XY,+X,+1,t(4;11)(q21;q23),+21[5]/46, X,-Y,t(4;11)(q21;q23),+mar[3]
329-13*	26	F	53.1		CD10+ CD19+ CD22+	VHR	46, XY,t(4;11)(q21;q23)[cp6]/49, XY,+X,+1,t(4;11)(q21;q23),+21[5]/46, X,-Y,t(4;11)(q21;q23),+mar[3]
**017-14**	22	M	39.5		CD10+ CD19+ CD22+	VHR	45, XY, −7,t(9;22)(q34;q11.2)[16]/46, XY[4] complement with translocation t(9;22) and monosomy 7 in sixteen of twenty cells analyzed; BCR/ABL1 transcript ratio: 0.37. Ratios>0.1
**280-13**	2	M	22.0	0.92 hypoploid	CD10+ CD19+ CD34+	STD	46, XY[4]
**116-13**	7	F	4.0	diploid	CD10+ CD19+ CD34+	STD	46, XX

* – have MLL-AF4 rearrangement

**Abbreviations:** *ALL:* acute lymphoblastic leukemia; *WBC*: white blood cell; *FISH*: fluorescent in situ hybridization; *STD*: standard risk; *VHR:* very high risk

Moreover, the overall shape of the dose-response curves obtained for different patient samples revealed an interesting pattern: the shape of the curves was more patient-specific rather than compound-specific. For example, in the samples from patients no. 1 and no. 5, we observed a significant increase in cell viability *vs*. vehicle treated sample at lower concentrations of dihydroartemisinin, clioquinol or parthenolide (see points above the dashed line, Figure 11A). This behavior was also evident in viability data from B-ALL cell lines (Figure 8). These results are consistent with the report that small or transient increases in icAMP can be anti-apoptotic and therefore, can support cell survival [2, 3]. Under identical conditions, the same compounds elicited no such effect in the samples obtained from patients no. 2 or no. 4. Another difference can be observed when the slopes of the dose-response curves are analyzed. It appears that in some patient samples, the slopes are steeper than in others. These data suggest that certain traits of a particular patient sample play a significant role in the response to different ICE compounds. It might be useful to correlate genotypic and phenotypic profiles of patient samples with these dose-response curve parameters. However, this will require further studies with larger numbers of patient samples with diverse genotypic and phenotypic profiles.

## DISCUSSION

For many years, researchers have been eager to develop methods to robustly modulate icAMP in cancer cells, and all known steps of the cAMP-related signaling pathway have been targeted [6]. Only recently, researchers have realized that icAMP can be also modulated by cyclic nucleotide efflux transporters. The finding that blocking cAMP efflux or down-regulation of efflux transporters can trigger cAMP elevation and downstream signaling [19] has opened new avenues for developing a novel class of anticancer therapeutics *via* inhibitors of cAMP efflux. Analysis of the proteins capable of cyclic nucleotide efflux also suggests notable possibilities.

The cAMP transporter ABCC4 has increased expression in blood cancer cells as compared to normal hematopoietic cells [26], and its expression decreases significantly upon cell differentiation [27]. ABC transporters are likewise upregulated in stem-like cells, which may suggest that these cells require active removal of cAMP or other structurally-related compounds in order to remain in a pluripotent state, and that the metabolic specificity of these cells requires active transport of certain metabolites [28]. These cancer stem cells are also associated with higher resistance to typical cancer therapeutics [29]. However, our results for ABCC4-expression did not define any clear correlations between cAMP efflux and ABCC4 expression in the cell lines. It is possible that other transporters capable of cAMP efflux (ABCC5 or ABCC11 [25, 30]) are present, or that there is a difference in the efflux capacity of different transporters. This may indicate that the ability to inhibit cAMP transport is substrate- or condition-dependent.

Of the active compounds identified in our screen for ICE, the majority were sesquiterpene lactones: parthenolide is derived from the feverfew (*Tanacetum parthenium*) plant; artemisinin and dihydroartemisinin are from *Artemisia annua*; and artesunate and artemether are semi-synthetic artemisinin derivatives. Cryptotanshinone is also plant-derived (*Salvia miotiorrhiza*), whereas clioquinol is an antimicrobial hydroxyquinolone derivative, and patulin is an antibiotic mycotoxin produced by *Aspergillus* and *Penicillium*. These compounds have been reported to exhibit anticancer activity in a number of model systems (Table 4), and the reported anticancer effects of these compounds were often similar to those seen in our assays (Table 5). However, none of the compounds has previously been reported to modulate cyclic nucleotide efflux.

**Table 4:** Previously reported anticancer activity of the compounds identified as ICE

Compound	Tested cancer types
**Patulin**	kidney[51], oral squamous cell carcinoma[52], glioblastoma[53], colon[54-56], colorectal[57], hematological[49,58], liver[59]
**Parthenolide**	melanoma[60,61], cervical[62,63], hematological[48,64,65], breast[66]
**Artesunate**	ovarian[67], cervical[68], Kaposi's sarcoma[69], squamous carcinoma[70], breast[71,72], liver[73], pancreatic[74,75], hematological[76-80], colorectal[79,81], neuroblatsoma[82], lung[79,83], osteosarcoma[84]
**Dihydroartemisinin**	ovarian[67,85], breast[86], liver[86,87], melanoma[88], pancreatic[89-93], lung[94-96], cervical[68], hematological[97-102], prostate[103], glioma[104], osteosarcoma[105]
**Clioquinol**	hematological[46,106-108], breast[109,110], cervical[111], prostate[112-114]
**Cryptotanshinone**	hematological[115,116], prostate[47,117,118], breast[47,119], melanoma[120,121], lung[121], rhabodomyosarcoma[47]

**Table 5:** Previously reported molecular/signaling mechanisms related to anticancer activity of the compounds identified as ICE

Compound	Cell cycle arrest	Anticancer effects	Potential mechanism(s) of action
**Parthenolide**	G1 [61,122], S [123]	↓VEGF expression & metastasis [61,124,125]; ↑p53 activation [48]; ↓cyclin D1 [61,122]	↓NF-κB & AP-1 activation [48,61,124,126,127]; ↓MAPK/ERK signaling [126,127]; ↓STAT signaling [128]; ↑ROS[48,65,128]
**Dihydroartemisinin**	G1 [90], G2 [87,101,105]	↓VEGF expression & angiogenesis [99,101,102]; ↓cyclin B, CDC25 [87]	↓NF-κB activation [90,105]; ↓MAPK/ERK, PI3K/Akt signaling [97,103]; ↑ROS [86,95,104]
**Artesunate**	G1 [70,129], S [81], G2 [79,84]	↓metastasis/migration [80,83]; ↓VEGF expression/angiogenesis [70,76,80]; ↓cyclins B & D1, Cdks 2, 4, 6[70]; ↑p21, p27 [70]	↓NFκB & AP-1 activation [83,130,131]; ↓nitric oxide, cAMP-mediated, Wnt/β-catenin, PI3K/Akt signaling [81,130,131]; ↑ROS [70,72,78]
**Cryptotanshinone**	G1 [120], G2 [120]	↓cyclin D1, Bcl-2 [47,115,116]; ↑p53, Chk1, Chk2 [120]	↓mTOR, STAT3 signaling [116,117]; ↓eIF-4E [47]
**Clioquinol**	G1 [110]	↓cyclin D1 [110]; ↑p21, p27, p53 [106]	↓NF-κB activation [108,112]; ionophore/chelator activity [106,108,112]; proteasome inhibition [107,109]
**Patulin**	G1 [132], G2 [57]	↓ERK1/2 activation [52,133]; ↑UPR[52,133]; ↑intracellular [Ca2+] [134]	↑ROS, DNA damage [57,59,132,133,135]; ↓glutathione [133,135]

Abbreviations: *VEGF:* vascular endothelial growth factor; *NF-κB:* nuclear factor kappa-B; *AP-1:* activator protein 1; *MAPK:* mitogen-activated protein kinase; *ERK:* extracellular signal-regulated kinases; *STAT:* signal transducer and activator of transcription; *ROS:* reactive oxygen species; *PI3K:* phosphatidylinositol-4,5-bisphosphate 3-kinase; *Akt:* protein kinase B; *mTOR:* mammalian target of rapamycin; *eIF-4E:* eukarytotic initiation factor 4E; *UPR:* unfolded protein response

Artemisinin, dihydroartemisinin, artesunate and artemether are currently used worldwide for the treatment of malaria. Artemether is a component of the drug Coartem manufactured by Novartis and approved by the US Food and Drug Administration in 2009. Several ongoing clinical trials of artemisinin derivatives to treat hepatocellular carcinoma, breast cancer, cervical intraepithelial neoplasia, as well as a trial evaluating “biological activity of oral clioquinol in patients with relapsed or refractory hematological malignancy” initiated by Dr. Mark Minden from Ontario Cancer Institute Princess Margaret Hospital in Canada, can be found on clinicaltrials.gov. Therefore, these drugs have the highest potential to be repurposed into current treatment regimens. Poor water-solubility and bioavailability of parthenolide prompted medical chemistry efforts to improve these characteristics [31]. However, since these drugs were identified from two relatively small libraries totaling ~3500 compounds, we envision that larger efforts to identify compounds capable of blocking cAMP efflux may lead to better drugs. This is especially important because of the unique signaling role of cAMP in apoptosis.

**Figure 12:**
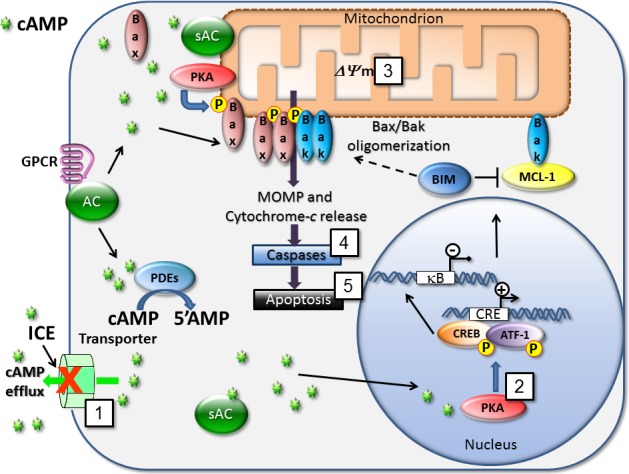
Mechanisms for cAMP-dependent regulation of cell death cAMP is produced by various pools of soluble and membrane-bound adenylyl cyclases (sAC and AC). Blocking cAMP efflux with ICE (1) triggers several signaling endpoints that are related to the modulation of pro-apoptotic and cell survival pathways. Here, we detected phosphorylation of CREB/ATF-1 (2), mitochondrial depolarization (3), effector caspases 3 and 7 activation (4), and apoptosis (5) in the form of annexin-V binding and cell membrane damage. Blocking adenylyl cyclase activity using selective inhibitor of soluble adenylyl cyclase (KH7) was sufficient to reduce effects of most potent compounds. Various molecular mechanisms are implicated in effects of cAMP on apoptosis induction. In AML cells pro-apoptotic protein Bim/BCL2L11 expression is up-regulated *via* CRE/CREB in a cAMP-dependent manner [2]. Induction of Bim represents a crucial event in the cAMP-induced apoptosis in murine T-cell lymphoma and human acute leukemia [9]. cAMP is also shown to inhibit MCL-1 protein transcription in human MM cells [8] or human B-precursor cells [18]. Relocalization of the cytosolic sAC pool toward mitochondria leads to PKA activation and phosphorylation of the pro-apoptotic protein Bax that activates mitochondrial apoptotic pathway [44]. Also, cAMP is implicated in a regulation of NF-κB action [32], which is a potential target in AML [45].

As a messenger, cAMP plays significant regulatory roles within cells. Multiple signaling mechanisms critical for leukemogenesis can be down-modulated by cAMP. At least two cAMP/PKA-related pathways can be involved in the induction of cAMP-dependent apoptosis in cancer cells: 1) the mitochondrial-mediated (intrinsic) pathway, and 2) modulation of the NF-κB signaling pathway (Figure 12). The pro-apoptotic intrinsic mechanism promoted by cAMP depends upon PKA [17], which can phosphorylate CREB, and results in transcription of the apoptotic activator Bim/BCL2L11 [2]. cAMP modulation of NF-κB can affect transcription of pro-survival genes [32]. Our data suggest that inhibition of cAMP transport by ICE was able to elicit many of these effects.

Targeting cancer through a “pathway-dependent approach” that consists of different means of elevating cAMP is considered a viable option for novel therapeutic development [6]. It appears that every potential step of the pathway, including cAMP synthesis, degradation, and downstream signaling, has been taken into consideration to stimulate icAMP accumulation [6, 33, 34]. Classically, cyclic nucleotide analogs or other cAMP-elevating agents have been used to treat hematological malignancies by slowing cell growth and differentiating cancer cells [14]. However, while modestly effective, these compounds exhibit toxicity in non-cancerous tissues [35-37]. Our data suggest that targeting cAMP efflux with small molecules could be an efficient way to raise icAMP in certain types of cancer, and this could potentially result in the development of a new class of pathway-specific therapeutics. Because increased cAMP efflux is not a typical trait of healthy cells, the identified ICE exhibited specificity toward leukemic cells. The relatively efficient targeting of cAMP transport in cancers would directly depend on cell- or patient-specific characteristics and efflux ability. We anticipate that identification of drugs that inhibit this transport could allow for selective targeting of cancers that capitalize on cAMP pathway modulation for survival, and this merits further investigation into the effects of ICE on multiple ABC transporters. The fact that the machinery responsible for apoptotic evasion by cAMP efflux can also potentially support the removal of structurally related chemotherapy drugs (e.g., ara-C), and thus may contribute to multidrug resistance [38-40], makes this work unusually promising.

## MATERIALS AND METHODS

### Ethics

All blood samples from healthy volunteers were obtained with written, informed consent per local institutional research guidelines according to the University of New Mexico Human Research Protections Office protocol 11-225. Bone marrow samples acquired from pediatric B-ALL patients were taken upon written, informed consent according to the University of New Mexico Human Research Protections Office protocol 05-435.

### Cells and reagents

Alexa Fluor^®^488 8-(6-aminohexyl) aminoadenosine 3′,5′-cyclicmonophosphate, bis(triethylammonium) salt (F-cAMP) (Life Technologies, cat. A35775). The VLA-4-specific probe 4-((N'-2-methylphenyl)ureido)-phenylacetyl-L-leucyl-L-aspartyl-L-valyl-L-prolyl-L-alanyl-L-alanyl-L-lysine-FITC (LDV-FITC) was synthesized at AIBioTech. The selective inhibitor of soluble adenylyl cyclase, KH7, was purchased from Cayman Chemical Company (cat. 330676-02-3). All other reagents and hit compounds for secondary assays were from Sigma-Aldrich.

Cell lines, purchased from ATCC and DSMZ, were cultured in RPMI-1640 medium supplemented with 2 mM L-glutamine, 100 U/ml penicillin-streptomycin and 10% heat-inactivated fetal bovine serum (FBS; 20% for MHH-Call3), hereafter referred to as cRPMI, and incubated in a humidified atmosphere with 5% CO_2_ at 37°C.

### PBMCs

Healthy PBMCs were obtained from volunteers. Mononuclear cells were purified using Mono-Poly resolving medium (MP Biomedicals, cat. 091698049) according to manufacturer's instructions. PBMCs were resuspended in cRPMI and kept on ice prior to use.

### Primary ALL patient samples

Bone marrow samples were acquired at diagnosis from pediatric B-ALL patients. Mononuclear cells were enriched by centrifugation in Ficoll-Paque (GE Healthcare, cat. 17-1440-02) and aliquoted for storage in liquid nitrogen until use. Samples were thawed in a 37°C water bath, resuspended in 20% FBS cRPMI, and centrifuged to remove freezing medium. Cells were resuspended in conditioned medium (DMEM, 10% FBS, 50 U/ml penicillin-streptomycin, 2 mM L-glutamine) from HS-5 stromal cell cultures.

### cAMP efflux assay

Cells were loaded with F-cAMP as described [41]. Briefly, cells were resuspended in a hypertonic solution containing 10% polyethylene glycol 1000, 0.5 M sucrose, and 250 μM F-cAMP in cRPMI without FBS, incubated 10 min at room temperature, washed, and then resuspended in hypotonic solution (60% cRPMI, 40% sterile water) for 2 min at room temperature to complete F-cAMP loading. Cells were resuspended in cRPMI and equilibrated for 2 h under normal culture conditions.

For testing cAMP efflux, an aliquot of loaded cells was kept at 4°C to serve as control, and the remaining cells were incubated in the presence of vehicle or compounds overnight. Samples were evaluated using Accuri C6 or BD FACScan flow cytometers (Becton Dickinson (BD)).

### High throughput screening (HTS)

Liquid handling was accomplished with a Biomek FX Multichannel system (Beckman-Coulter, Fullerton, CA, USA) and/or Biotek Multiflo system (Winooski, VT, USA). F-cAMP-loaded U937 cells (2000/well) were seeded into 384-well polypropylene plates (Greiner Bio-One 784201, Monroe, NC, USA). Cells were treated with compounds (10 μM final concentration) from the Prestwick Chemical Library (Illkirch, France) or SPECTRUM Collection (MicroSource, Gaylordsville, CT, USA) delivered by pintool (V&P Scientific, San Diego, CA), at a final DMSO concentration of 1%. Plates were foil-sealed with AlumaSeal 384™ (Excel Scientific, Victorville, CA, USA), and incubated inverted overnight. Sample plates were analyzed by CyAn flow cytometers (Beckman-Coulter) configured with HyperCyt high-throughput auto-sampler systems (IntelliCyt, Albuquerque, NM, USA) using 488 nm excitation to assess FL-1 (530/40) MFI levels.

Data were analyzed with HyperView software (IntelliCyt) and time-gated to determine data from each well. Wells with ≥ 50 events were evaluated for FL-1 MFI values. Samples with MFI values ≥ 2 standard deviations above the plate mean negative control values were considered “hit” compounds.

### Compound validation

To validate sample data and decrease the number of false-positive hits, compounds were assayed in a high-throughput dose response assay. Plates were set up as in the HTS, with the exception that the plate formats contained 10-well dose responses for each hit compound, at final concentrations ranging from 30 μM to 4 nM.

For B-ALL cell lines, tests of hit compound inhibition of cAMP efflux were conducted as in the HTS, with compounds in 10-well dose responses ranging from 100 μM - 1.53 nM, at cell densities of 5000/well.

The dose response data were normalized for percent response based on sample FL-1 MFI values in comparison to untreated F-cAMP-loaded cells kept at 4°C overnight or at time = 0, and were fitted by GraphPad Prism 5 software (GraphPad Software, La Jolla, CA, USA) for sigmoidal dose response with constrained hill slope = 1. Compounds with S-shaped dose response curves and EC_50_ values less than 3 μM were selected for secondary assays.

### Detection of CREB/AFT-1 phosphorylation in response to ICE

Cells were suspended at 10^6^ cells/ml in cRPMI and incubated for 1 hour at 37°C with 20 μM ICE compounds or forskolin (positive control). Negative control samples were treated with DMSO at equal volume. Cells were fixed with 20x volume of pre-warmed 1x solution BD Phosflow^TM^ Lyse/Fix Buffer (cat. 558049), incubated at 37°C for 10 minutes, permeabilized with BD Phosflow^TM^ Perm buffer II (cat. 558052), and incubated on ice for 30 minutes. Permeabilized cells were then washed and stained with BD Phosflow™ Alexa Fluor^®^ 488 Mouse Anti-CREB (pS133) / ATF-1 (pS63) clone J151-21 (cat. 558435) according to the manufacturer's instructions.

### Kinetic analysis of VLA-4 deactivation

Kinetic analysis of the binding and dissociation of the LDV-FITC probe was described previously [42]. Briefly, flow cytometric data were acquired at 37°C while the samples were stirred continuously with a stir bar (Bel-Art Products). First, U937 cells transfected with a non-desensitizing mutant of the Formyl Peptide Receptor FPR1 [43] were analyzed for 30-120 s to establish a baseline. The FPR1 mutant triggers VLA-4 activation, which persists for hundreds of seconds, allowing for integrin deactivation to be detected. Next, the LDV-FITC was added and acquisition was re-established. For cell activation, the high affinity FPR-specific agonist (N-formyl-L-methionyl-L-leucyl-L-phenylalanyl-L-phenylalanine, fMLFF) was added at a saturating concentration (100 nM), and acquisition was re-established. Finally, 40 μM of ICE, forskolin (positive control) or vehicle (negative control) were added. Acquisition was re-established, and data were acquired continuously for up to 1024 s.

The concentration of the LDV-FITC probe used in deactivation experiments (4 nM) was below the dissociation constant (K_d_) for its binding to resting VLA-4 (low affinity state, K_d_ ~12 nM), and above the K_d_ for physiologically activated VLA-4 (high affinity state, K_d_ ~1-2 nM). Therefore, the transition from low to high affinity state led to increased binding of the probe. This was detected as an increase in the median fluorescence intensity (MFI). VLA-4 deactivation led to the dissociation of the probe and decreased MFI [21].

### Viability

Cell viability was determined with the CellTiter-Glo^®^ Luminescent Cell Viability Assay (Promega). Greiner 655083 had 100 μL cRPMI +/− 4×10^4^ cells/well, and dose response curves of compounds ranged from 100 μM to 1.53 nM (1% DMSO). Wells with DMSO-only served as negative controls. Samples were incubated overnight, and the assay was completed according to the manufacturer's protocol. Plates were analyzed using a Victor^3^V™ 1420 Multilabel Counter (Perkin Elmer).

For primary ALL patient samples, media consisted of 50% cRPMI, 50% conditioned DMEM from HS-5 stromal cell cultures and compounds. Cell densities ranged from 28,000-68,000 cells/well, and plates were foil-sealed prior to incubation.

### Determination of cell viability by 7-AAD exclusion

Cells treated with ICE and control samples were incubated with 1 μg/ml of 7-AAD for 20-60 minutes at 4°C on a rotator in the dark, and analyzed by flow cytometry according to manufacturer instructions. Gates were set on 7-AAD negative and positive events based on histograms. Percent of 7-AAD negative events is shown.

### Apoptosis

Apoptosis was determined using MultiCyt^®^ 4-Plex Apoptosis Screening Kit (IntelliCyt, cat. 90053). The kit provides four different apoptosis endpoints that include effector caspases 3 and 7 activation, phosphatidylserine surface expression, mitochondrial membrane depolarization and cell membrane integrity. The activation of caspases is detected using NucView™ 488 Caspase3/7-specific substrate that exhibits increase in fluorescence upon cleavage by activated enzyme. Surface phosphatidylserine is detected by the binding of labelled annexin-V. Another fluorescent dye accumulates in intact mitochondria and upon mitochondrial membrane depolarization it leaks into the cytoplasm and exhibits a decrease in fluorescence. Cell membrane damage is detected by the uptake of a proprietary DNA intercalating agent analogous to 7-aminoactinomycin D.

384-well plates (Greiner 784201), pre-loaded with 5 μL cRPMI were stamped using a pintool with 100 nl of DMSO-solubilized compounds from the 100-fold concentrated stock solution. Next, 5 μL U937 cells (2 × 10^6^/ml in cRPMI) were added to wells and mixed, and plates were foil-sealed. Compounds (Table 1) were tested in dose response at final concentrations ranging from 15.2 nM to 100 μM (1% DMSO final). Vehicle (DMSO) was used as a negative control. After 24 h incubation under culture conditions, the IntelliCyt MultiCyt 4-Plex Apoptosis Screening Kit was used according to manufacturer's protocol. The data were acquired using iQue Screener Platform (IntelliCyt). Singlet cell populations were analyzed for individual measures of apoptosis, and gates were set based on control histograms.

### Quantitation of ABCC4

ABCC4 transporters in fixed B-ALL cell lines were enumerated by flow cytometry using: primary mouse anti-human ABCC4 or IgG1 negative isotype-matched control (Abcam^®^, Cambridge, MA, USA), FITC-conjugated AffiniPure F(ab')2 of goat anti-mouse IgG secondary antibody (Jackson Laboratories, West Grove, PA, USA), and Quantum™ Simply Cellular^®^ anti-human IgG calibration beads (Bangs Laboratories, Fishers, IN, USA) according to manufacturers' protocols. Post-labeling, all samples were analyzed for FL-1 MFI. The calibration beads were used to generate a linear regression, which associated MFI to the antibody-binding capacity of the beads. This regression was used to calculate the number of antibody binding sites (ABS) per cell sample. For each cell type, the calculated ABS for isotype control samples were subtracted from ABS of the ABCC4 samples to determine specific binding sites.

### Data analysis

The kinetic data were converted to MFI *versus* time using FCSQuery software developed by Dr. Bruce Edwards (University of New Mexico). Analysis of apoptosis was done using ForeCyt software (IntelliCyt). Curve fits and statistics were done using GraphPad Prism software version 5.01 (GraphPad Software), as described in figure captions.
